# Biomarkers for Comorbidities Modulate the Activity of T-Cells in COPD

**DOI:** 10.3390/ijms22137187

**Published:** 2021-07-02

**Authors:** Kaschin Jamal Jameel, Willem-Jakob Gallert, Sarah D. Yanik, Susanne Panek, Juliane Kronsbein, David Jungck, Andrea Koch, Jürgen Knobloch

**Affiliations:** 1 Medical Clinic III for Pneumology, Allergology and Sleep Medicine, Bergmannsheil University Hospital, Ruhr-University Bochum, Bürkle-de-la-Camp-Platz 1, 44789 Bochum, Germany; kaschin.jamaljameel@rub.de (K.J.J.); willem-jakob.gallert@gmx.de (W.-J.G.); Sarah.Yanik@gmx.de (S.D.Y.); susanne.panek@st-vinzenz-hospital.de (S.P.); juliane.kronsbein@bergmannsheil.de (J.K.); 2 Department of Internal Medicine II, Pneumology, Allergology and Respiratory Medicine, Bethel Teaching Hospital, 12207 Berlin, Germany; David.Jungck@BethelNet.de; 3 Pyhrn-Eisenwurzen-Klinikum Steyr, Klinik für Pneumologie, Lehrkrankenhaus der Uniklinik Linz, Sierninger Str. 170, 4400 Steyr, Austria; Andrea.Koch2@ooeg.at; 4 Ludwig-Maximilians-University of Munich (LMU) and DZL (German Center of Lung Science), 81377 Munich, Germany

**Keywords:** COPD, comorbidities, T-cells

## Abstract

In smoking-induced chronic obstructive pulmonary disease (COPD), various comorbidities are linked to systemic inflammation and infection-induced exacerbations. The underlying mechanisms are unclear but might provide therapeutic targets. T-cell activity is central in systemic inflammation and for infection-defense mechanisms and might be influenced by comorbidities. Hypothesis: Circulating biomarkers of comorbidities modulate the activity of T-cells of the T-helper type 1 (Th1) and/or T-cytotoxic type 1 (Tc1). T-cells in peripheral blood mononuclear cells (PBMCs) from non-smokers (NS), current smokers without COPD (S), and COPD subjects (total *n* = 34) were ex vivo activated towards Th1/Tc1 and were then stimulated with biomarkers for metabolic and/or cardiovascular comorbidities (Brain Natriuretic Peptide, BNP; chemokine (C-C motif) ligand 18, CCL18; C-X3-C motif chemokine ligand 1, CX3CL1; interleukin-18, IL-18) or for asthma- and/or cancer-related comorbidities (CCL22; epidermal growth factor, EGF; IL-17; periostin) each at 10 or 50 ng/mL. The Th1/Tc1 activation markers interferon-γ (IFNγ), tumor necrosis factor-α (TNFα), and granulocyte-macrophage colony-stimulating factor (GM-CSF) were analyzed in culture supernatants by Enzyme-Linked Immunosorbent Assay (ELISA). Ex-vivo activation induced IFNγ and TNFα without differences between the groups but GM-CSF more in S vs. NS. At 10 ng/mL, the different biomarkers increased or reduced the T-cell activation markers without a clear trend for one direction in the different categories of comorbidities or for the different T-cell activation markers. At 50 ng/mL, there was a clear shift towards suppressive effects, particularly for the asthma— and cancer-related biomarkers and in cells of S and COPD. Comorbidities might suppress T-cell immunity in COPD. This could explain the association of comorbidities with frequent exacerbations.

## 1. Introduction

Chronic obstructive pulmonary disease (COPD) is mainly induced by tobacco smoking. It is a systemic inflammatory disease associated with various comorbidities that have a negative impact on prognosis and progression. The development and progression of comorbidities might be triggered by systemic inflammation [1]. Comorbidities are associated with the frequency of exacerbations, another major trigger of progression [2]. COPD subjects have an increased susceptibility to bacterial and viral infections, both of which are major causes of exacerbations [3]. 

Cluster of differentiation (CD) 4+ and CD8+ T-cells of the T-helper type 1 (Th1) or T-cytotoxic type 1 (Tc1), respectively, are central in the systemic inflammation in COPD and for the immune responses to those pathogens that are frequently found in COPD subjects [4,5]. Mechanistic links between T-cell activity and comorbidities are largely unknown but might provide insights into the association of comorbidities with exacerbations and suggest corresponding drug targets. Many comorbidities are characterized by circulating biomarkers that are often cytokines or chemokines whose receptors are expressed on T-cells or on accessory immune cells. In response to airway infections with most COPD-characteristic pathogens, inactive naive and memory T-cells become activated towards Th1 or Tc1 effector T-cells. During this activation process, the T-cells become recruited from the circulation to the local sites of infection. This implicates that comorbidities might influence T-cell activation via their circulating biomarkers, a possible mechanistic link between systemic inflammation, comorbidities and exacerbations. Therefore, this study aimed to investigate the impact of systemic biomarkers for frequent COPD comorbidities on T-cell activity.

Following the clearance of a pathogen a subset of T-cells differentiates into memory T-cells, which downregulate their effector program but maintain the ability to rapidly reactivate upon re-infection [6]. T-cell exhaustion describes an altered differentiation stage of memory T-cells with an increased expression of multiple inhibitory receptors like programmed cell death protein-1 (PD-1) and a reduced capacity to regain effector functions upon re-infection. Exhausted T-cells are heterogeneous and have been primarily described as a consequence of persistent exposure to antigens and inflammatory factors in diseases with chronic viral infection and chronic inflammation but also in cancer [7]. Exhausted T-cells do not lose full responsiveness to re-infections but they respond with a reduced effectiveness, which might result in an ineffective or delayed clearance of the pathogen [6].

Chronic inflammation and infection are prominent in COPD and several cancer types are common co-morbidities (see below). In line with this, there is evidence for the presence of CD8+ and CD4+ T-cells in the lung and in the circulation with a reduced capacity to respond to acute infections [8,9]. The CD8+ T-cells show an increased expression of the inhibitory receptor PD-1 in COPD, but it is still a matter of debate if they match the criteria of exhausted T-cells [10]. On the other hand, the recruitment process of CD8+ T-cells is disturbed in COPD, leading to a massive intrapulmonary infiltration of Tc1 cells [11,12]. Thus, in response to acute respiratory infections a surplus of T-cells might arrive in the lung that, however, have a reduced capacity to respond to the pathogens. As a consequence, the pathogen clearance is disturbed but local inflammation becomes increased. From this, we hypothesized that the persistent exposure of T-cells to the inflammation-associated biomarkers of comorbidities in COPD might also reduce the effector activity of T-cells. Obviously, these biomarkers characterize the corresponding diseases also in the absence of COPD. Therefore, possible effects of the biomarkers on T-cell activity in subjects without COPD would have relevance for the associated diseases as well, a point that was also considered in the design of the present study.

Frequent comorbidities in COPD are metabolic and heart diseases, particularly type 2 diabetes, arteriosclerosis, coronary artery disease, and heart failure, all of which are linked to each other [13,14]. There is evidence from retrospective studies that comorbidities and particularly diabetes and heart failure are associated with frequent exacerbations in COPD [2]. The adipokine C-X3-C motif chemokine ligand 1 (CX3CL1, Fractalkine) and interleukin-18 (IL-18) both are associated with type 2 diabetes and are increased in the plasma of respective patients [15,16]. CX3CL1 and IL-18 both are further associated with coronary artery disease (CAD) and are increased in the plasma of CAD patients [17,18]. Chemokine (C-C motif) ligand 18 (CCL18) is also increased in the plasma of CAD patients and is associated with CAD severity [19]. CX3CL1 and CCL18, both are particularly increased in patients with refractory unstable angina, a symptom of CAD [18,20]. CX3CL1 is further associated with Carotid artery stenosis, a common coincidence with CAD and a further common consequence of arteriosclerosis, and is increased in the plasma of respective patients [21]. Brain Natriuretic Peptide (BNP) is a strong biomarker for heart failure, for which CAD is a major risk factor [22]. Its plasma levels are increased in patients with acute heart failure [23]. Serum CX3CL1, IL-18, and CCL18 are increased in stable COPD and are associated with disease progression and severity [24,25,26]. An association between BNP and stable COPD has, to our knowledge, not yet been observed. 

Further common comorbidities in COPD are asthma, lung cancer, and non-pulmonary cancer types like bladder, breast, colorectal, ovarian, and prostate cancer, for example [27,28]. Asthma and lung cancer might also be associated with frequent exacerbations in COPD [2]. An increased serum periostin level is a strong marker for type 2 asthma [29] and might also have prognostic significance for non-small cell lung cancer (NSCLC) and various non-pulmonary cancer types including those mentioned [30,31,32]. Serum IL-17 is increased in obesity-associated asthma and might be indicative of severe phenotypes [33,34]. Serum CCL22 is increased in breast cancer and is indicative of progression and severity [35]. Serum epidermal growth factor (EGF) is suitable to distinguish NSCLC from healthy benign lung pathologies [36]. Increased serum EGF concentrations are also discussed as putative biomarkers for various non-pulmonary cancer types including gastrointestinal cancers [37]. Serum periostin and EGF but not IL-17 and CCL22 are increased in stable COPD [38,39,40,41].

We hypothesized that these biomarkers of comorbidities suppress the activation process of T-cells towards Th1/Tc1, thereby contributing to a deficit in immune responses to infections and to exacerbations in COPD. We used the human primary peripheral blood mononuclear cell (PBMC) culture model with ex-vivo activation of T-cells to address this question (1) because the circulating biomarkers likely have contact with the circulating immune cells at the beginning of recruitment and activation in vivo, and (2) because the influence of the biomarkers on T-cell activation might depend on accessory cells, for example, if T-cells do not functionally express the corresponding receptors. With the PBMC model, the influence of accessory cells is considered. Analysis parameters were markers for Th1/Tc1 activation, interferon-γ (IFNγ, a key marker), tumor necrosis factor-α (TNFα), and granulocyte-macrophage colony-stimulating factor (GM-CSF). We compared the data between non-smokers, current smokers without airway disease, and COPD subjects in order to analyze for specific effects of smoking or COPD in this context and to gain first evidence for possible effects in the biomarker-associated diseases in the absence of COPD.

## 2. Results

### 2.1. Cytokine Release in Response to T-Cell Activation

**Figure 1:**
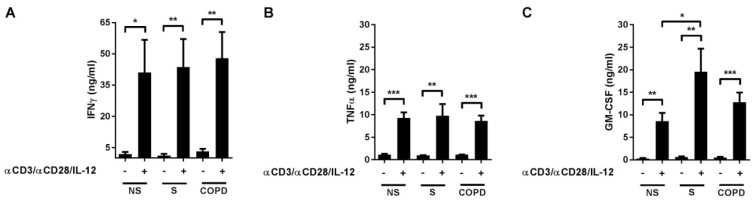
T-cell activation towards Th1/Tc1 induced the release of IFNγ, TNFα, and GM-CSF in peripheral blood mononuclear cells (PBMCs) of non-smokers (NS), current smokers without respiratory symptoms (S) and COPD subjects. Peripheral blood mononuclear cells (PBMCs; 10^6^ cells/mL) of NS (*n* = 10), S (*n* = 11), and COPD (*n* = 13) were stimulated with anti-CD3 and anti-CD28 antibodies (each at 500 ng/mL) and with IL-12 (10 ng/mL). After 72h, INFγ (**A**), TNFα (**B**) and GM-CSF (**C**) concentrations were measured in the cell culture supernatants by enzyme-linked immunosorbent assay (ELISA). The cytokine levels of the controls without T-cell activating reagents are artificial because they were below the detection limit of the ELISA. Data are presented as mean ± SEM. Differences between activated cells and non-activated controls within a group were analyzed with paired *t*-tests, differences between the groups were analyzed with one-way analysis of variance (ANOVA, *p* < 0.0001 in C) and post hoc Bonferroni test. *, *p* < 0.05; **, *p* < 0.01; ***, *p* < 0.001.

The activation of T-cells in PBMCs with anti-CD3 and anti-CD28 antibodies and IL-12 induced the release of the Th1 and Tc1 activation marker IFNγ (Figure 1A). Additionally, TNFα and GM-CSF, both of which are associated with Th1/Tc1 responses, were induced (Figure 1B,C). The induction of GM-CSF was higher in cells of current smokers without respiratory symptoms (S) compared to non-smokers (NS) (Figure 1C). IFNγ and TNFα were not different between the groups (Figure 1A,B). We did not find correlations of IFNγ, TNFα, or GM-CSF concentrations after T-cell activation to age, pack-years, lung function parameters (forced expiratory volume in one second, FEV1 (% pred.), FEV1/forced vital capacity, FEV1/FVC (%)) or to differential blood count parameters (monocytes (% whole blood count, WBC), lymphocytes (% WBC), neutrophils (% WBC), eosinophils (% WBC)), neither by analyzing all subjects together nor by analyzing the groups S or COPD separately (data not shown). 

We next tested for cytotoxic effects of CX3CL1, IL-18, CCL18, BNP, periostin, CCL22, IL-17, and EGF in this model. We did not find effects on the numbers of trypan blue positive cells for concentrations up to 50 ng/mL for each recombinant protein (data not shown). Therefore, we used 10 and 50 ng/mL in the following approaches.

### 2.2. CX3CL1 Increased IFNγ, TNFα, and GM-CSF Release of T-Cells

**Figure 2:**
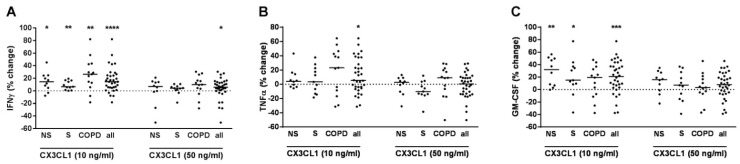
CX3CL1 further increased INFγ, TNFα and GM-CSF release from PBMCs with activated T-cells. PBMCs from nonsmokers (NS; *n* = 10), current smokers without respiratory symptoms (S; *n* = 11) and chronic obstructive pulmonary disease subjects (COPD; *n* = 13) were stimulated with anti-CD3 and anti-CD28 antibodies (each at 500 ng/mL) and with IL-12 (10 ng/mL). After 30 min, recombinant CX3CL1 was added at 10 or 50 ng/mL. After 72h, INFγ (**A**), TNFα (**B**) and GM-CSF (**C**) concentrations were measured in the cell culture supernatants by ELISA. Data were calculated as % change versus PBMCs that were stimulated with anti-CD3/anti-CD28 antibodies and IL-12. Data are presented as scatter with median. The effects of CX3CL1 on the cytokines were analyzed by Wilcoxon-signed rank test vs. a hypothetical value of 0 (= no change). *, *p* < 0.05; **, *p* < 0.01; ***, *p* < 0.001; ***, *p* < 0.001.

When all subjects were analyzed together independent from disease status, CX3CL1 concentration-dependently further increased IFNγ, TNFα, and GM-CSF in PBMCs pre-treated with anti-CD3 and anti-CD28 antibodies and with IL-12 (Figure 2A–C). After grouping according to COPD and smoking status, this effect was without differences between NS, S, and COPD for IFNγ (Figure 2A). For TNFα, this effect was not observed in any subgroup (Figure 2B). For GM-CSF, this effect was observed in NS and S without differences but not in the COPD subgroup (Figure 2C). We did not find any correlation of the increase of IFNγ, TNFα or GM-CSF to the demographic, lung function or blood count parameters (data not shown). In culture supernatants of PBMCs that were not pre-treated with T-cell activating reagents but were stimulated with CX3CL1, the concentrations of IFNγ, TNFα or GM-CSF were almost always below the detection limit of the ELISA at the conditions used (data not shown). 

### 2.3. IL-18 Increased IFNγ but Reduced TNFα and GM-CSF Release of T-Cells

**Figure 3:**
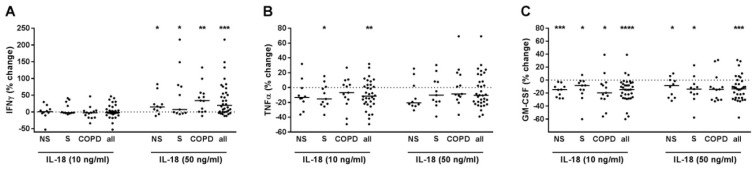
IL-18 modulated IFNγ, TNFα and GM-CSF release from PBMCs with activated T-cells. PBMCs from nonsmokers (NS; *n* = 10), current smokers without respiratory symptoms (S; *n* = 11) and chronic obstructive pulmonary disease subjects (COPD; *n* = 13) were stimulated with anti-CD3 and anti-CD28 antibodies (each at 500 ng/mL) and with IL-12 (10 ng/mL). After 30 min, recombinant IL-18 was added at 10 or 50 ng/mL. After 72 h, INFγ (**A**), TNFα (**B**) and GM-CSF (**C**) concentrations were measured in the cell culture supernatants by ELISA. Data were calculated as % change versus PBMCs that were stimulated with anti-CD3/anti-CD28 antibodies and IL-12. Data are presented as scatter with median. The effects of IL-18 on the cytokines were analyzed by one-sample test vs. a hypothetical value of 0 (= no change). *, *p* < 0.05; **, *p* < 0.01; ***, *p* < 0.001; ****, *p* < 0.0001.

IL-18 concentration-dependently further increased IFNγ (Figure 3A) but decreased TNFα, and GM-CSF in PBMCs pre-treated with anti-CD3 and anti-CD28 antibodies and with IL-12 (Figure 3B,C). After subgrouping, the effects of IL-18 on IFNγ and GM-CSF were without difference between NS, S, and COPD (Figure 3A,C). The reducing effect of IL-18 on TNFα was only observed in S. (Figure 3B). We did not find any correlation to the demographic, lung function or blood count parameters (data not shown). In culture supernatants of PBMCs that were not pre-treated with T-cell activating reagents but were stimulated with IL-18, the concentrations of IFNγ, TNFα or GM-CSF were almost always below the detection limit of the ELISA at the conditions used (data not shown).

### 2.4. CCL18 Concentration-Dependently Increased or Decreased the Cytokine Release of T-Cells

**Figure 4:**
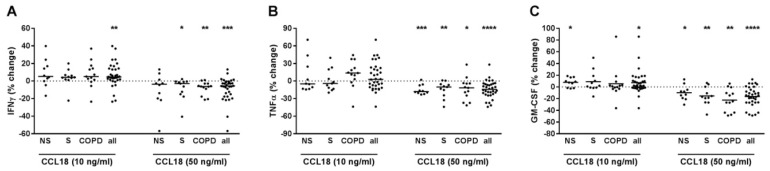
CCL18 concentration-dependently modulated INFγ, TNFα and GM-CSF release from PBMCs with activated T-cells. PBMCs from nonsmokers (NS; *n* = 10), current smokers without respiratory symptoms (S; *n* = 11) and chronic obstructive pulmonary disease subjects (COPD; *n* = 13) were stimulated with anti-CD3 and anti-CD28 antibodies (each at 500 ng/mL) and with IL-12 (10 ng/mL). After 30 min, recombinant CCL18 was added at 10 or 50 ng/mL. After 72h, INFγ (**A**), TNFα (**B**) and GM-CSF (**C**) concentrations were measured in the cell culture supernatants by ELISA. Data were calculated as % change versus PBMCs that were stimulated with anti-CD3/anti-CD28 antibodies and IL-12. Data are presented as scatter with median. The effects of CCL18 on the cytokines were analyzed by one-sample test vs. a hypothetical value of 0 (= no change). *, *p* < 0.05; **, *p* < 0.01; ***, *p* < 0.001; ****, *p* < 0.0001.

At 10 ng/mL, CCL18 further increased IFNγ and GM-CSF but not TNFα in PBMCs pre-treated with anti-CD3 and anti-CD28 antibodies and with IL-12 (Figure 4A–C). After grouping, the effect on IFNγ was not observed in any group (Figure 4A). The effect on GM-CSF was observed in NS but not in S or COPD (Figure 4C). We did not find any correlation to the demographic, lung function or blood count parameters (data not shown). At 50 ng/mL, CCL18 reduced all three cytokines (Figure 4A–C). After grouping, the effects on IFNγ were observed in S and COPD but not in NS (Figure 4A). The effects on TNFα and GM-CSF were observed in all groups without differences (Figure 4B,C). In the COPD group, the reducing effect of CCL18 at 50 ng/mL on TNFα correlated positively to age (r^2^ = 0.333; *p* = 0.038) and negatively to FEV1/FVC (%) (r^2^ = 0.328; *p* = 0.04). The suppressing effect on GM-CSF correlated negatively to the monocyte content in the blood (% WBC) of COPD subjects (r^2^ = 0.352; *p* = 0.033). We did not find any correlations for IFNγ (data not shown). In culture supernatants of PBMCs that were not pre-treated with T-cell activating reagents but were stimulated with CCL18, the concentrations of IFNγ, TNFα or GM-CSF were almost always below the detection limit of the ELISA in the conditions used (data not shown).

### 2.5. BNP Reduced TNFα and GM-CSF Release of T-Cells

**Figure 5:**
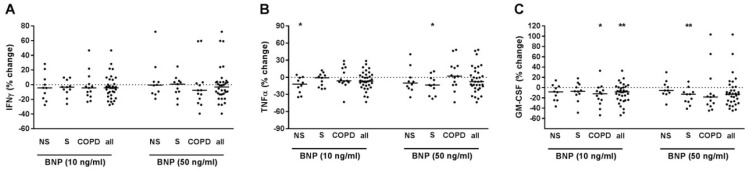
BMP reduced TNFα and GM-CSF release from PBMCs with activated T-cells. PBMCs from nonsmokers (NS; *n* = 10), current smokers without respiratory symptoms (S; *n* = 11) and chronic obstructive pulmonary disease subjects (COPD; *n* = 13) were stimulated with anti-CD3 and anti-CD28 antibodies (each at 500 ng/mL) and with IL-12 (10 ng/mL). After 30 min, recombinant BNP was added at 10 or 50 ng/mL. After 72 h, INFγ (**A**), TNFα (**B**) and GM-CSF (**C**) concentrations were measured in the cell culture supernatants by ELISA. Data were calculated as % change versus PBMCs that were stimulated with anti-CD3/anti-CD28 antibodies and IL-12. Data are presented as scatter with median. The effects of BNP on the cytokines were analyzed by Wilcoxon-signed rank test vs. a hypothetical value of 0 (= no change). *, *p* < 0.05; **, *p* < 0.01.

When all study subjects were analyzed together, BNP concentration-dependently reduced GM-CSF but did not modulate IFNγ or TNFα in PBMCs pre-treated with anti-CD3 and anti-CD28 antibodies and with IL-12 (Figure 5A–C). After grouping, BNP concentration-dependently reduced TNFα in NS and S but not in COPD (Figure 5B) and GM-CSF in S and COPD but not in NS (Figure 5C). BNP did not modulate IFNγ in any group (Figure 5A). The suppressive effect of BNP on GM-CSF correlated positively to neutrophils (% WBC) (r^2^ = 0.375, *p* = 0.026) and negatively to lymphocytes (% WBC) (r^2^ = 0.35, *p* = 0.033). We did not find correlations for IFNγ or TNFα. In culture supernatants of PBMCs that were not pre-treated with T-cell activating reagents but were stimulated with BNP, the concentrations of IFNγ, TNFα or GM-CSF were almost always below the detection limit of the ELISA in the conditions used (data not shown).

### 2.6. Periostin Did Not Modulate IFNγ, TNFα or GM-CSF Release of T-Cells from Current Smokers without Respiratory Symptoms and from COPD Subjects

**Figure 6:**
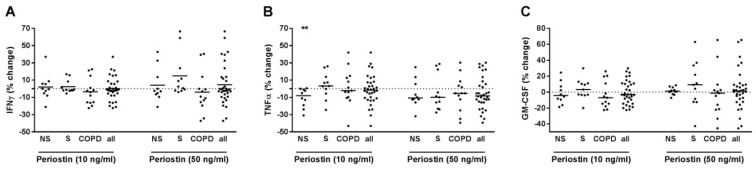
Periostin did not modulate IFNγ, TNFα or GM-CSF release from PBMCs with activated T-cells in S or COPD. PBMCs from nonsmokers (NS; *n* = 10), current smokers without respiratory symptoms (S; *n* = 11) and chronic obstructive pulmonary disease subjects (COPD; *n* = 13) were stimulated with anti-CD3 and anti-CD28 antibodies (each at 500 ng/mL) and with IL-12 (10 ng/mL). After 30 min, recombinant periostin was added at 10 or 50 ng/mL. After 72 h, INFγ (**A**), TNFα (**B**) and GM-CSF (**C**) concentrations were measured in the cell culture supernatants by ELISA. Data were calculated as % change versus PBMCs that were stimulated with anti-CD3/anti-CD28 antibodies and IL-12. Data are presented as scatter with median. The effects of periostin on the cytokines were analyzed by Wilcoxon-signed rank test vs. a hypothetical value of 0 (= no change). **, *p* < 0.01.

When analyzing all subjects together, periostin did not modulate IFNγ, TNFα or GM-CSF in PBMCs with pre-treatment with anti-CD3 and anti-CD28 antibodies and IL-12 (Figure 6A–C). After grouping, periostin reduced TNFα exclusively in pre-treated PBMCs of NS in a concentration-dependent manner (Figure 6B). We did not find any correlation of the effects of periostin on the cytokines with demographic, lung function or blood count parameters (data not shown). In culture supernatants of PBMCs that were not pre-treated with T-cell activating reagents but were stimulated with periostin, the concentrations of IFNγ, TNFα or GM-CSF were almost always below the detection limit of the ELISA in the conditions used (data not shown).

### 2.7. IL-17 Suppressed GM-CSF Release of T-Cells

**Figure 7:**
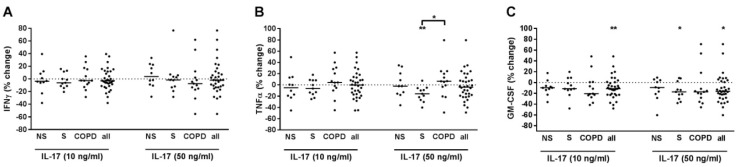
IL-17 suppressed TNFα and GM-CSF release from PBMCs with activated T-cells in current smokers without COPD. PBMCs from nonsmokers (NS; *n* = 10), current smokers without respiratory symptoms (S; *n* = 11) and chronic obstructive pulmonary disease subjects (COPD; *n* = 13) were stimulated with anti-CD3 and anti-CD28 antibodies (each at 500 ng/mL) and with IL-12 (10 ng/mL). After 30 min, recombinant IL-17 was added at 10 or 50 ng/mL. After 72 h, INFγ (**A**), TNFα (**B**) and GM-CSF (**C**) concentrations were measured in the cell culture supernatants by ELISA. Data were calculated as % change versus PBMCs that were stimulated with anti-CD3/anti-CD28 antibodies and IL-12. Data are presented as scatter with median. The effects of IL-17 on the cytokines were analyzed by Wilcoxon-signed-rank test vs. a hypothetical value of 0 (= no change). Comparisons between the groups were made by Kruskal-Wallis test (*p* = 0.039 in B) with post hoc Dunn’s test. *, *p* < 0.05; **, *p* < 0.01.

When analyzing all subjects together, IL-17 reduced GM-CSF but did not modulate IFNγ and TNFα in PBMCs pre-treated with anti-CD3 and anti-CD28 antibodies and with IL-12 (Figure 7A–C). After grouping, this effect was exclusively observed in S (Figure 7C). IL-17 concentration-dependently reduced TNFα in S but not in NS and COPD (Figure 7B). We did not find any correlation of the effects of IL-17 to demographic, lung function or blood count parameters (data not shown). In culture supernatants of PBMCs that were not pre-treated with T-cell activating reagents but were stimulated with IL-17, the concentrations of IFNγ, TNFα or GM-CSF were almost always below the detection limit of the ELISA in the conditions used (data not shown).

### 2.8. CCL22 Suppressed IFNγ, TNFα and GM-CSF Release of T-Cells

**Figure 8:**
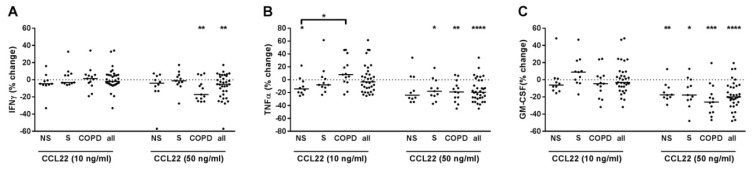
CCL22 reduced IFNγ, TNFα, and GM-CSF release from PBMCs with activated T-cells. PBMCs from non-smokers (NS; *n* = 10), current smokers without respiratory symptoms (S; *n* = 11) and chronic obstructive pulmonary disease subjects (COPD; *n* = 13) were stimulated with anti-CD3 and anti-CD28 antibodies (each at 500 ng/mL) and with IL-12 (10 ng/mL). After 30 min, recombinant CCL22 was added at 10 or 50 ng/mL. After 72 h, INFγ (**A**), TNFα (**B**) and GM-CSF (**C**) concentrations were measured in the cell culture supernatants by ELISA. Data were calculated as % change versus PBMCs that were stimulated with anti-CD3/anti-CD28 antibodies and IL-12. Data are presented as scatter with median. The effects of CCL22 on the cytokines were analyzed by one-sample test vs. a hypothetical value of 0 (= no change). Comparisons between the groups were made with one-way ANOVA and post hoc Bonferroni test. *, *p* < 0.05; **, *p* < 0.01; ***, *p* < 0.001; ****, *p* < 0.0001.

CCL22 concentration-dependently reduced IFNγ, TNFα and GM-CSF in PBMCs pre-treated with anti-CD3 and anti-CD28 antibodies and with IL-12 (Figure 8A–C). After grouping, CCL22 reduced IFNγ only in COPD (Figure 8A) but TNFα and GM-CSF in all three groups (Figure 8B,C). The effect of CCL22 at 10 ng/mL on TNFα was higher in NS compared to COPD (Figure 8B). When analyzing all subjects together, the suppressive effect of CCL22 at 50 ng/mL on IFNγ correlated negatively and that at 10 ng/mL on TNFα correlated positively with FEV1 (% pred.). We did not find correlations for GM-CSF (data not shown). In culture supernatants of PBMCs that were not pre-treated with T-cell activating reagents but were stimulated with CCL22, the concentrations of IFNγ, TNFα or GM-CSF were almost always below the detection limit of the ELISA at the conditions used (data not shown).

### 2.9. EGF Modulated IFNγ and GM-CSF Release of T-Cells

**Figure 9:**
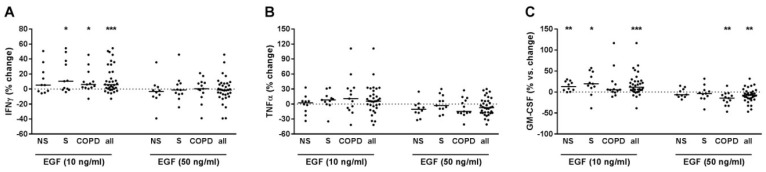
EGF modulates the release of IFNγ and GM-CSF from PBMCs with activated T-cells. PBMCs from nonsmokers (NS; *n* = 10), current smokers without respiratory symptoms (S; *n* = 11) and chronic obstructive pulmonary disease subjects (COPD; *n* = 13) were stimulated with anti-CD3 and anti-CD28 antibodies (each at 500 ng/mL) and with IL-12 (10 ng/mL). After 30 min, recombinant EGF was added at 10 or 50 ng/mL. After 72 h, INFγ (**A**), TNFα (**B**) and GM-CSF (**C**) concentrations were measured in the cell culture supernatants by ELISA. Data were calculated as % change versus PBMCs that were stimulated with anti-CD3/anti-CD28 antibodies and IL-12. Data are presented as scatter with median. The effects of EGF on the cytokines were analyzed by one-sample test vs. a hypothetical value of 0 (= no change). *, *p* < 0.05; **, *p* < 0.01; ***, *p* < 0.001.

At 10 ng/mL, EGF increased IFNγ and GM-CSF but not TNFα in PBMCs pre-treated with anti-CD3 and anti-CD28 antibodies and with IL-12 (Figure 9A–C). After grouping, the EGF effect on IFNγ was exclusively observed in S and COPD (Figure 9A). The effect on GM-CSF was observed in NS and S but not in COPD (Figure 9C). We did not find correlations to the demographic, lung function or blood count parameters. At 50 ng/mL EGF reduced GM-CSF but did not modulate IFNγ and TNFα. After subgrouping, the effect was exclusively observed in COPD. When all subjects were analyzed together, the suppressive effect on GM-CSF correlated negatively to FEV1 (% pred.) (r^2^ = 0.169; *p* = 0.016) and to FEV1/FVC (%) (r^2^ = 0.133; *p* = 0.029). The negative correlation to FEV1 (% pred.) was also observed in the COPD group (r^2^ = 0.331; *p* = 0.040). In culture supernatants of PBMCs that were not pre-treated with T-cell activating reagents but were stimulated with EGF, the concentrations of IFNγ, TNFα or GM-CSF were almost always below the detection limit of the ELISA in the conditions used (data not shown).

## 3. Discussion

INFγ and TNFα release of T-cells after activation towards Th1/Tc1 in PBMCs was not influenced by the smoking status of the subjects or by COPD and was also not associated with COPD-related lung function parameters. For IFNγ, this matches to previous data with isolated CD4+ T-cells [9]. GM-CSF release from the T-cells of current smokers, however, was increased compared to non-smokers. Smoking induces GM-CSF and granulocyte levels in the lung, which contributes to smoking-induced lung inflammation [42,43,44]. Epithelial cells and/or macrophages have been discussed as a primary source of these increased GM-CSF levels, but our data indicate that recruited T-cells might contribute to this pathology. We did not observe increased GM-CSF production in T-cells of COPD subjects. This could be explained by the fact that about 60% of the COPD subjects in our cohort were ex-smokers. Moreover, our data did not provide indication for an exhaustion of T-cells regarding the responsiveness to T cell-receptor and co-receptor stimulation specifically in COPD.

To address the question of mechanistic links between co-morbidities, systemic inflammation and infection-induced exacerbations, we tested for effects of the respective circulating biomarkers on T-cell activity and T-cell activation towards Th1/Tc1 in the PBMC culture model. The model considers the presence of accessory cells that influence the T-cell activation process and the possible influence of co-morbidity biomarkers at recruitment from the circulation to the draining lymph nodes in response to acute infections in vivo.

Acute infections in the last two months before sampling was exclusion criterion. Thus, the presence of T-cells that have already been activated in vivo in response to acute infections were almost excluded in the PBMC cultures. When using cells of COPD subjects, our experimental model, therefore, reflects the activation of T-cells in stable COPD in response to an acute infection (that can cause an exacerbation) in the presence of comorbidities. The acute infection was mimicked by adding the T-cell activating reagents to the culture and the comorbidities were represented by adding the respective biomarkers.

**Figure 10:**
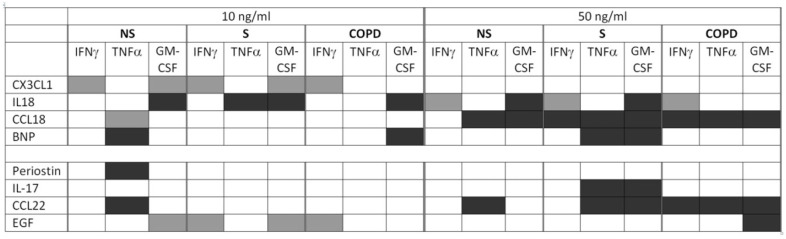
Summary of the biomarker effects on Th1/Tc1 cytokines in subject groups. Statistically significant effects from Figures 2–9 are shown. Light grey, up-regulation; dark grey, down-regulation. Biomarker concentrations are given in the top row. Group 1 includes biomarkers for metabolic and cardiovascular diseases (CXCL1, IL-18, CCL18, BNP), group 2 in-cludes biomarkers for asthma and cancer (Periostin, IL-17, CCL22, EGF). NS, non-smokers, S, current smokers without respiratory symptoms, COPD, chronic obstructive pulmonary disease.

We have seen various smoking-, COPD-, and concentration-dependent effects of the biomarkers on the Th1 activation process. The effects were in a range of up to 30% increase or decrease of T-cell activity in terms of cytokine production and were not associated with cytotoxicity according to trypan blue staining in pre-experiments. Independent of disease and smoking status of the subjects, the effects of a single biomarker concentration may vary in strength and also in direction between IFNγ, TNFα, and GM-CSF (Figure 10). For example, IL-18 at 50 ng/mL upregulated IFNγ but reduced GM-CSF in NS. This further confirms that the reductions of cytokine levels are not based on general cytotoxic effects of the recombinant proteins. Furthermore, some of the effects that have been observed here match to previous studies. The increasing effect of IL-18 on IFNγ is in line with data that have suggested IL-18 to be an enhancer of IFNγ-based Th1 cell activity in cooperation with IL-12 [45]. CCL22 reduced the activity of T-cells polarized towards Th1/Tc1. This matches to data that have shown a reduced Th1 effector function of infiltrating CD4+ and CD8+ cells after exposure to CCL22 [46]. In animal studies, the inhibition of the NPRA signaling pathway, a BNP target, resulted in an increased TNFα expression, which suggests respective suppressive effects of BNP [47]. Our data confirm this conclusion and indicate that T-cells might contribute to the mechanism.

There are two general limitations of this model that have to be considered before discussing the data in the clinical context. First, the cytokines analyzed might have been released from other cells than T-cells, exclusively or as well. This issue can be neglected here because in the control experiments without addition of T-cell activating reagents the concentrations of all three target cytokines were almost below the detection limit of the ELISA in the presence or absence of the biomarkers. Second, we cannot answer the question whether the biomarker effects on T-cells were the result of a direct response or if they were mediated by accessory cells. This issue can be neglected for the discussion regarding possible links between co-morbidities, systemic inflammation and exacerbations, the primary goal of the study. However, it gains more importance when trying to deduce possible therapeutic targets from these data. Therefore, the differential expression of the receptors for these biomarkers should be considered. With the exception of the BNP receptor Natriuretic peptide receptor A (NPR-A), which is expressed on accessory monocytes [48], the primary receptors for all other biomarkers used here are expressed on CD4+ and/or CD8+ T-cells and also on accessory cells of the PBMC fraction. The CX3CL1 receptor CX3CR1 is expressed on CD8+ T-cells, Th1-cells, monocytes and natural killer (NK) cells [18,49]. The IL-18 receptor (IL18R) is expressed on T-cells, B-cells, NK cells and on dendritic cells (DCs) [50,51]. CCR8, the primary CCL18 receptor, is expressed at low levels on Th1 cells, CD8+ T-cells and NK cells [52]. The EGF receptor (EGFR) and the periostin receptors αV/β3 and αV/β5 integrins are expressed on T-cells and on monocytes [53,54]. CCR4, the CCL22 receptor can be found on Th1 and NK cells and on DCs [55,56,57]. The heterodimer IL17RA/RC, the IL17 receptor, is abundantly expressed on monocytes and B-cells but in low levels also on inactive CD3+ T-cells [58]. Therefore, the effects of BNP on T-cell activity are indirect and might be mediated by monocytes, whereas the effects of the other biomarkers on T-cell activity might be direct or indirect or a combination of both. A third limitation is that the study was not powered to subgroup the COPD subjects for comorbidities. According to the study design, adding the biomarkers to the culture represents the respective comorbidity in this experimental model. A possible exposition of the cells with the biomarkers in vivo before sampling might have influenced their response to the reagents in culture. Nonetheless, the broad variation of comorbidities in the limited *n*-number of COPD subjects did not allow a corresponding stratification of the data.

At 10 ng/mL, the different biomarkers increased or reduced the T-cell activation markers without a clear trend for one direction in the different categories of comorbidities or for the different T-cell activation markers. However, increasing the biomarker concentrations clearly resulted in an increase of the suppressive effects, particularly for the biomarkers associated with asthma and cancer (Figure 10). Indeed, at 50 ng/mL, an up-regulation was only observed for IFNγ in response to the diabetes and CAD marker IL-18, but a reduction for at least one of the three T-cell activity markers in response to all other biomarkers except CX3CL1 and periostin. We interpret a reduced T-cell activity caused by a comorbidity-biomarker in our experimental model as a putative mechanistic reason for an increased possibility to get or prolong an exacerbation in COPD with the respective comorbidity. This is because a reduced T-cell-dependent infection defense might result in a delayed clearance of the pathogen. In this context, it is important to observe the respective biomarker effects in the COPD group but it is irrelevant if there are differences to healthy subjects or active smokers without respiratory symptoms. Whether these T-cells with the reduced activity share characteristics of exhausted T-cells remains to be investigated.

Provided that the severity of a comorbidity is associated with an increase of the respective biomarkers in the circulation, our data indicate that the progression of co-morbidities in COPD is associated with a reduced activity of Th1/Tc1 cells in response to acute infections. This would suggest a reduced capacity of the immune response to acute respiratory tract infections and could contribute to the mechanisms underlying the association of comorbidities with frequent exacerbations.

With two exceptions, we did not detect statistically significant differences when comparing the three subject groups, NS, S, and COPD, for the biomarker effects on T-cell activity. Nevertheless, we think that there is some evidence for an influence of active smoking and systemic COPD pathology on the biomarker effects. It is noteworthy that at 50 ng/mL statistically significant suppressive effects were more often observed in S and COPD than in NS, particularly again for those biomarkers associated with asthma and cancer (Figure 10). In NS, only TNFα was reduced by CCL22. In S, TNFα and GM-CSF both were reduced by CCL22 and by IL-17. In COPD, we did not find effects of IL-17, but all three T-cell activity markers were reduced by CCL22, and GM-CSF was also reduced by EGF. This provides first evidence that systemic consequences of active smoking as well as of the COPD systemic pathology might influence the effects of asthma and cancer related co-morbidity biomarkers on T-cell activity. To a lesser extent this also applies to the biomarkers of cardiovascular co-morbidities, CCL18 and BNP. In summary, we carefully conclude that smoking and the COPD pathology might enhance the suppressive effects of the biomarkers for co-morbidities on T-cell immunity. However, because of the low numbers of significant differences between the groups, this conclusion requires further investigation. The observation that some of the biomarkers also modulate the T-cell activity in cells of healthy never smokers provides first indication that the diseases that are associated with the biomarkers might also influence T-cell immunity in the absence of COPD. However, compared to the COPD group, the overall trend was less pronounced towards suppressive effects.

The biomarker effects could have been influenced by the activation status of the T-cells in the samples before starting the culture despite our exclusion criteria. There is evidence for an increase in the number of activated circulating CD8+ T-cells and for dysregulated regulatory T-cells also in the absence of an acute infection in stable COPD, however, the data are controversial [59,60]. Our controls did not indicate any significant T-cell activity in the cultured PBMCs in the absence of T-cell activation reagents in any group (Figure 1). We cannot exclude the presence of CD4+ or CD8+ T-cells in the cultures that have been deactivated by regulatory T-cells in vivo before sampling.

This study adds another part to the understanding of the complex systemic molecular pathology that underlies the increased susceptibility to respiratory infections in COPD. In contrast to the local innate immune cells that show an overactivation in response to respiratory pathogens in COPD [11,61], the activation process of circulating innate and adaptive immune cells appears to be rather suppressed. We have previously shown that systemic defects in Toll-like receptor signaling prevent the full activation of T-cells and monocytes in response to respiratory bacteria [9,62,63]. Here, we add the information that the suppression of the Th1-immunity might be amplified by co-morbidities in COPD.

## 4. Material and Methods

### 4.1. Study Subjects

The study population consisted of three groups: 10 healthy non-smokers (≥20 years of nonsmoking; <1 pack-year) (NS); 11 current smokers (≥10 pack-years) without respiratory symptoms (S); and 13 patients with stable COPD, five current and eight former smokers (≥10 packyears; Global Initiative for Chronic Obstructive Lung Disease (GOLD) stages II—IV) (Table 1). COPD was diagnosed according to the criteria recommended by the National Institutes of Health and according to GOLD standard. The following comorbidities were recorded in the COPD subjects at sampling: hypothyroidism (*n* = 2), scoliosis (*n* = 1), hypertension (*n* = 2), type II diabetes and hypertension (*n* = 1), history of cervix cancer (*n* = 1), gastroesophageal reflux disease (*n* = 1), peripheral arterial disease (*n* = 1), multiple (aortic and mitral valve insufficiency, arthrosis, hypertension, gastroesophageal reflux disease, glaucoma, gout, osteoporosis; *n* = 1), none (*n* = 3). Sample size calculation was based on preliminary experiments with activated T-cells in PBMCs (*n* = 3 per group) for the biomarker CX3CL1 and the analysis parameter IFNγ (Th1/Tc1 activation marker). With α < 0.05 and a power (1-β) of 0.8 the number of patients to be included was nine per group. We increased the number to 10–13 per group in order to compensate for putative errors in recruiting or in sample preparation, handling or analysis. Inclusion and exclusion criteria were defined according to previous studies [9]: age: ≥40 years; no use of oral corticosteroids or immunosuppressive treatments and no report of acute infection (bacterial, viral, or parasitic) 2 months before sampling. From 37 subjects recruited, three subjects were excluded because they did not match the in— or exclusion criteria or because of sample limitation resulting in missing endpoints after analyses. The study was conducted according to the guidelines of the Declaration of Helsinki, and approved by the Institutional Ethics Committee of the Ruhr-University Bochum, (4257-12, 06.07.2012), Bochum, Germany”.

### 4.2. Isolation of Peripheral Blood Mononuclear Cells (PBMCs) 

The isolation of PBMCs from the peripheral blood of donors was performed via Ficoll gradient sedimentation according to established protocols [9,64]. Cell counts and vitality tests were done by trypan blue (Sigma, München, Germany, cat# T8154) staining as described before [9]. Trypan blue positive cells were below 10%. 2.5x10^5^ PBMCs per approach were cultured overnight in 250 µL RPMI1640 medium (Sigma, Munich, Germany) supplemented with 10% (*v*/*v*) fetal calf serum (FCS), 2 mM L-glutamine, 100 U/mL penicillin and 100 μg/mL streptomycin (all from Sigma) at 5% CO_2_ and 37° C as described before [65]. T-cell activation and differentiation towards Th1/Tc1 in PBMCs was done as described before [63]. Briefly, PBMCs were stimulated with agonistic monoclonal anti-CD3 and anti-CD28 antibodies (500 ng/mL each; BD Bioscience, Heidelberg, Germany, cat# 555336, cat# 555725) and 10 ng/mL recombinant human IL-12 (R&D Systems, Wiesbaden, Germany, cat# 219-IL-005). The biomarkers for comorbidities were added 30 min after T-cell activation (each at 10 ng/mL and 50 ng/mL): recombinant human periostin, EGF, CCL18, IL-18, CCL22, IL-17, CX3CL1, (all from R&D Systems, Wiesbaden, Germany; cat# 3548-F2, 236E6, 394-PA, B003-5, 336-MD/8F, 317-ILB-050, 365-FR/CF) and BNP (Biomol GmbH, Hamburg, Germany, cat# 87386). After 72 h, the culture supernatants were collected and analyzed for cytokine concentrations.

### 4.3. Enzyme-Linked Immunosorbent Assay for IFNγ, TNFα and GM-CSF

IFNγ, TNFα and GM-CSF concentrations in the supernatants of cultured PBMCs were measured by ELISA according to the manufacturer’s instructions (R&D Systems, Minneapolis, MN; cat# DY285, DY210, DY215) and as described in [66]. 

### 4.4. Statistical Analysis

Statistical analyses were performed to investigate whether the biomarkers for comorbidities influence the T-cell activation and if there are differences between the groups. The distribution of the data were analyzed by histogram analysis. Cytokine concentration data were presented as mean ± standard error of the mean (SEM). Data that show the influence of the biomarkers on cytokine release were normalized, expressed as % change (versus T-cell activation without biomarker stimulation), and presented as scatter with median. Paired *t*-test, one-sample *t*-test, and Wilcoxon-signed-rank test were used to analyze for differences between approaches within a group. One-way analysis of variance (ANOVA) with post hoc Bonferroni test or Kruskal-Wallis with post hoc Dunns test were used to analyze for differences between the study groups. Correlations of cytokine concentrations in the cell culture supernatants to age, pack-years, lung function parameters (FEV1, % pred.; FEV1/FVC, %) and differential blood count parameters (monocytes, % of whole blood count (WBC); lymphocytes, % WBC; neutrophils, % WBC; eosinophils, % WBC) were investigated by linear regression analysis. For all tests, a *p*-value below 0.05 was considered statistically significant. All tests were done with GraphPad Prism 5.01 (Graph Pad Software; San Diego, CA, USA).

## Figures and Tables

**Table 1:** Demographics of patients.

Parameter	NS	S	COPD
N	10	11	13
Age, y	56.6 ± 3.6	56.7 ± 2.6	61.9 ± 2.3
Gender, m:f	2:8	4:7	7:6
FEV_1_, % pred.	104.8 ± 4.7	105.5 ± 4.3	45.9 ± 2.9 ***
FEV_1_/FVC, %	79.4 ± 1.8	79.3 ± 1.3	45 ± 3.1 ***
FVC, % pred.	109.1 ± 5.5	108.9 ± 4.2	84.5 ± 4.3 **
Pack-Years	0	50.7 ± 10.8	53.7 ± 7.5
Monocytes, %WBC	6.3 ± 0.5	9.2 ± 0.7 ^##^	4.5 ± 0.5 ^§§^
Lymphocytes, %WBC	37.5 ± 4.7	34.3 ± 3.5	27.7 ± 2.1
Neutrophils, %WBC	55.2 ± 4.9	56.5 ± 4.0	67.3 ± 2.6
Eosinophils, %WBC	1.0 ± 0.3	0.9 ± 0.4	0.5 ± 0.2

NS, non-smoker; S, current smoker without chronic obstructive pulmonary disease (COPD) (≥10 pack-years); COPD, chronic obstructive pulmonary disease. COPD subjects were Global Initiative for Chronic Obstructive Lung Disease (GOLD) stages II to IV. FEV_1_, forced expiratory volume in one second; FVC, forced vital capacity; WBC, whole blood count; Data are given as mean ± standard error of the mean (SEM); ** *p* < 0.01; *** *p* <0.001 versus S and NS; ^##^ *p* < 0.01 vs. NS; ^§§^ *p* < 0.01 vs. S.

## Data Availability

The original data, the statistical analyses and the data that were cited as “not shown” can be obtained from the corresponding author upon request.

## References

[1] Pelgrim C.E., Peterson J.D., Gosker H.R., Schols A.M., van Helvoort A., Garssen J., Folkerts G., Kraneveld A.D. (2019). Psychological co-morbidities in COPD: Targeting systemic inflammation, a benefit for both?. Eur. J. Pharmacol..

[2] Westerik J.A.M., Metting E.I., Van Boven J.F.M., Tiersma W., Kocks J.W.H., Schermer T.R. (2017). Associations between chronic comorbidity and exacerbation risk in primary care patients with COPD. Respir. Res..

[3] Rangelov K., Sethi S. (2014). Role of Infections. Clin. Chest Med..

[4] Paats M., Bergen I., Hoogsteden H., van der Eerden M., Hendriks R. (2011). Systemic CD4+ and CD8+ T-cell cytokine profiles correlate with GOLD stage in stable COPD. Eur. Respir. J..

[5] Roberts M.E.P., Higgs B., Brohawn B.P., Pilataxi B.F., Guo X., Kuziora M., Bowler R.P., White W.I. (2015). CD4+ T-Cell Profiles and Peripheral Blood Ex-Vivo Responses to T-Cell Directed Stimulation Delineate COPD Phenotypes. Chronic Obstr. Pulm. Dis. J. COPD Found..

[6] Wherry E.J., Kurachi M. (2015). Molecular and cellular insights into T cell exhaustion. Nat. Rev. Immunol..

[7] Blank C.U., Haining W.N., Held W., Hogan P.G., Kallies A., Lugli E., Lynn R.C., Philip M., Rao A., Restifo N.P. (2019). Defining ‘T cell exhaustion’. Nat. Rev. Immunol..

[8] McKendry R.T., Spalluto C.M., Burke H., Nicholas B., Cellura M.D., Al-Shamkhani A., Staples K., Wilkinson T.M.A. (2016). Dysregulation of Antiviral Function of CD8+T Cells in the Chronic Obstructive Pulmonary Disease Lung. Role of the PD-1–PD-L1 Axis. Am. J. Respir. Crit. Care Med..

[9] Knobloch J., Schild K., Jungck D., Urban K., Müller K., Schweda E.K.H., Rupp J., Koch A. (2011). The T-Helper Cell Type 1 Immune Response to Gram-Negative Bacterial Infections Is Impaired in COPD. Am. J. Respir. Crit. Care Med..

[10] Curtis J.L. (2016). At the Checkpoint: Lung CD8+T Cells, Respiratory Viruses, and Chronic Obstructive Pulmonary Disease. Am. J. Respir. Crit. Care Med..

[11] Barnes P.J. (2016). Inflammatory mechanisms in patients with chronic obstructive pulmonary disease. J. Allergy Clin. Immunol..

[12] Zhu X., Gadgil A.S., Givelber R., George M.P., Stoner M.W., Sciurba F., Duncan S.R. (2009). Peripheral T Cell Functions Correlate with the Severity of Chronic Obstructive Pulmonary Disease. J. Immunol..

[13] Gayle A., Dickinson S., Poole C., Pang M., Fauconnot O., Quint J.K. (2019). Incidence of type II diabetes in chronic obstructive pulmonary disease: A nested case–control study. NPJ Prim. Care Respir. Med..

[14] Trinkmann F., Saur J., Borggrefe M., Akin I. (2019). Cardiovascular Comorbidities in Chronic Obstructive Pulmonary Disease (COPD)—Current Considerations for Clinical Practice. J. Clin. Med..

[15] Shah R., Hinkle C.C., Ferguson J., Mehta N.N., Li M., Qu L., Lu Y., Putt M.E., Ahima R.S., Reilly M.P. (2011). Fractalkine Is a Novel Human Adipochemokine Associated With Type 2 Diabetes. Diabetes.

[16] Zaharieva E., Kamenov Z., Velikova T., Tsakova A., El-Darawish Y., Okamura H. (2018). Interleukin-18 serum level is elevated in type 2 diabetes and latent autoimmune diabetes. Endocr. Connect..

[17] Blankenberg S., Rupprecht H.J., Poirier O., Bickel C., Smieja M., Hafner G., Meyer J., Cambien F., Tiret L. (2003). Plasma Concentrations and Genetic Variation of Matrix Metalloproteinase 9 and Prognosis of Patients With Cardiovascular Disease. Circulation.

[18] Damås J.K., Boullier A., Waehre T., Smith C., Sandberg W.J., Green S., Aukrust P., Quehenberger O. (2005). Expression of Fractalkine (CX3CL1) and its Receptor, CX3CR1, Is Elevated in Coronary Artery Disease and Is Reduced During Statin Therapy. Arter. Thromb. Vasc. Biol..

[19] Versteylen M.O., Manca M., Joosen I.A., Schmidt D.E., Das M., Hofstra L., Crijns H.J., Biessen E.A., Kietselaer B.L. (2016). CC chemokine ligands in patients presenting with stable chest pain: Association with atherosclerosis and future cardiovascular events. Neth. Hear. J..

[20] Kraaijeveld A., de Jager S., de Jager W., Prakken B., McColl S., Haspels I., Putter H., van Berkel T., Nagelkerken L., Jukema J. (2007). CC Chemokine Ligand-5 (CCL5/RANTES) and CC Chemokine Ligand-18 (CCL18/PARC) Are Specific Markers of Refractory Unstable Angina Pectoris and Are Transiently Raised During Severe Ischemic Symptoms. Circulation.

[21] Stolla M., Pelisek J., Von Brühl M.-L., Schäfer A., Barocke V., Heider P., Lorenz M., Tirniceriu A., Steinhart A., Bauersachs J. (2012). Fractalkine Is Expressed in Early and Advanced Atherosclerotic Lesions and Supports Monocyte Recruitment via CX3CR1. PLoS ONE.

[22] Velagaleti R.S., Vasan R.S. (2007). Heart Failure in the Twenty-First Century: Is it a Coronary Artery Disease or Hypertension Problem?. Cardiol. Clin..

[23] Maisel A.S., Krishnaswamy P., Nowak R.M., Mccord J., Hollander J.E., Duc P., Omland T., Storrow A.B., Abraham W.T., Wu A.H. (2002). Rapid Measurement of B-Type Natriuretic Peptide in the Emergency Diagnosis of Heart Failure. N. Engl. J. Med..

[24] Hao W., Li M., Zhang C., Zhang Y., Du W. (2020). Increased levels of inflammatory biomarker CX3CL1 in patients with chronic obstructive pulmonary disease. Cytokine.

[25] Imaoka H., Hoshino T., Takei S., Kinoshita T., Okamoto M., Kawayama T., Kato S., Iwasaki H., Watanabe K., Aizawa H. (2008). Interleukin-18 production and pulmonary function in COPD. Eur. Respir. J..

[26] Sin D.D., Miller B.E., Duvoix A., Man S.F.P., Zhang X., Silverman E.K., Connett J.E., Anthonisen N.A., Wise R., Tashkin D. (2011). Serum PARC/CCL-18 Concentrations and Health Outcomes in Chronic Obstructive Pulmonary Disease. Am. J. Respir. Crit. Care Med..

[27] Gagnat A.A., Gjerdevik M., Gallefoss F., Coxson H.O., Gulsvik A., Bakke P. (2017). Incidence of non-pulmonary cancer and lung cancer by amount of emphysema and airway wall thickness: A community-based cohort. Eur. Respir. J..

[28] Maselli D.J., Hanania N.A. (2018). Asthma COPD overlap: Impact of associated comorbidities. Pulm. Pharmacol. Ther..

[29] Izuhara K., Conway S.J., Moore B., Matsumoto H., Holweg C.T.J., Matthews J.G., Arron J.R. (2016). Roles of Periostin in Respiratory Disorders. Am. J. Respir. Crit. Care Med..

[30] González-González L., Alonso J. (2018). Periostin: A Matricellular Protein with Multiple Functions in Cancer Development and Progression. Front. Oncol..

[31] Hong L.-Z., Wei X.-W., Chen J.-F., Shi Y. (2013). Overexpression of periostin predicts poor prognosis in non-small cell lung cancer. Oncol. Lett..

[32] Xu C.-H., Wang W., Lin Y., Qian L.-H., Zhang X.-W., Wang Q.-B., Yu L.-K. (2016). Diagnostic and prognostic value of serum periostin in patients with non-small cell lung cancer. Oncotarget.

[33] Agache I., Ciobanu C., Agache C., Anghel M. (2010). Increased serum IL-17 is an independent risk factor for severe asthma. Respir. Med..

[34] Chen K., Pociask D.A., McAleer J.P., Chan Y.R., Alcorn J.F., Kreindler J.L., Keyser M.R., Shapiro S.D., Houghton A.M., Kolls J.K. (2011). IL-17RA Is Required for CCL2 Expression, Macrophage Recruitment, and Emphysema in Response to Cigarette Smoke. PLoS ONE.

[35] Jafarzadeh A., Fooladseresht H., Minaee K., Bazrafshani M.R., Khosravimashizi A., Nemati M., Mohammadizadeh M., Mohammadi M.M., Ghaderi A. (2014). Higher circulating levels of chemokine CCL22 in patients with breast cancer: Evaluation of the influences of tumor stage and chemokine gene polymorphism. Tumor Biol..

[36] Blanco-Prieto S., De Chiara L., Rodríguez-Girondo M., Vázquez-Iglesias L., Rodríguez-Berrocal F.J., Fernandez-Villar A., Botana-Rial M.I., De La Cadena M.P. (2017). Highly Sensitive Marker Panel for Guidance in Lung Cancer Rapid Diagnostic Units. Sci. Rep..

[37] Jiang L., Hochwald S., Deng S., Zhu Y., Tan C., Zhong Q., Zhou Y., Zhao H., Huang H. (2017). Evaluation of EGF, EGFR, and E-cadherin as potential biomarkers for gastrointestinal cancers. Front. Lab. Med..

[38] Carpaij O.A., Muntinghe F.O.W., Wagenaar M.B., Habing J.W., Timens W., Kerstjens H.A.M., Nawijn M., Kunz L.I.Z., Hiemstra P.S., Tew G.W. (2018). Serum periostin does not reflect type 2-driven inflammation in COPD. Respir. Res..

[39] Kim V., Cornwell W.D., Oros M., Durra H., Criner G.J., Rogers T.J. (2015). Plasma Chemokine signature correlates with lung goblet cell hyperplasia in smokers with and without chronic obstructive pulmonary disease. BMC Pulm. Med..

[40] Loza M.J., Watt R., Baribaud F., Barnathan E.S., Rennard S.I. (2012). Systemic inflammatory profile and response to anti-tumor necrosis factor therapy in chronic obstructive pulmonary disease. Respir. Res..

[41] Zou Y., Chen X., Liu J., Zhou D.B., Kuang X., Xiao J., Yu Q., Lu X., Li W., Xie B. (2017). Serum IL-1β and IL-17 levels in patients with COPD: Associations with clinical parameters. Int. J. Chronic Obstr. Pulm. Dis..

[42] Chung K.F., Caramori G., Adcock I.M., Di Stefano A. (2014). Cytokine inhibition in the treatment of COPD. Int. J. Chronic Obstr. Pulm. Dis..

[43] Vlahos R., Bozinovski S., Chan S.P.J., Ivanov S., Lindén A., Hamilton J.A., Anderson G.P. (2010). Neutralizing Granulocyte/Macrophage Colony–Stimulating Factor Inhibits Cigarette Smoke–induced Lung Inflammation. Am. J. Respir. Crit. Care Med..

[44] Vlahos R., Bozinovski S., Hamilton J.A., Anderson G. (2006). Therapeutic potential of treating chronic obstructive pulmonary disease (COPD) by neutralising granulocyte macrophage-colony stimulating factor (GM-CSF). Pharmacol. Ther..

[45] Yasuda K., Nakanishi K., Tsutsui H. (2019). Interleukin-18 in Health and Disease. Int. J. Mol. Sci..

[46] Bischoff L., Alvarez S., Dai D.L., Soukhatcheva G., Orban P.C., Verchere C.B. (2015). Cellular Mechanisms of CCL22-Mediated Attenuation of Autoimmune Diabetes. J. Immunol..

[47] Vellaichamy E., Kaur K., Pandey K.N. (2007). Enhanced activation of pro-inflammatory cytokines in mice lacking natriuretic peptide receptor-A. Peptides.

[48] Glezeva N., Collier P., Voon V., Ledwidge M., McDonald K., Watson C., Baugh J. (2013). Attenuation of Monocyte Chemotaxis—A Novel Anti-inflammatory Mechanism of Action for the Cardio-protective Hormone B-Type Natriuretic Peptide. J. Cardiovasc. Transl. Res..

[49] Jones B.A., Beamer M., Ahmed S. (2010). Fractalkine/CX3CL1: A Potential New Target for Inflammatory Diseases. Mol. Interv..

[50] Gracie J.A., Robertson S.E., McInnes I. (2003). Interleukin-18. J. Leukoc. Biol..

[51] Xu D., Chan W.L., Leung B.P., Hunter D., Schulz K., Carter R.W., McInnes I., Robinson J.H., Liew F.Y. (1998). Selective Expression and Functions of Interleukin 18 Receptor on T Helper (Th) Type 1 but not Th2 Cells. J. Exp. Med..

[52] Soler D., Chapman T.R., Poisson L.R., Wang L., Cote-Sierra J., Ryan M., McDonald A., Badola S., Fedyk E., Coyle A.J. (2006). CCR8 expression identifies CD4 memory T cells enriched for FOXP3+ regulatory and Th2 effector lymphocytes. J. Immunol..

[53] Huang S., Endo R.I., Nemerow G.R. (1995). Upregulation of integrins alpha v beta 3 and alpha v beta 5 on human monocytes and T lymphocytes facilitates adenovirus-mediated gene delivery. J. Virol..

[54] Tsuchiya K., Jo T., Takeda N., Al Heialy S., Siddiqui S., Shalaby K.H., Risse P.-A., Maghni K., Martin J.G. (2010). EGF receptor activation during allergic sensitization affects IL-6-induced T-cell influx to airways in a rat model of asthma. Eur. J. Immunol..

[55] Nanki T., Lipsky P.E. (2000). Lack of correlation between chemokine receptor and Th1/Th2 cytokine expression by individual memory T cells. Int. Immunol..

[56] Purandare A., Somerville J. (2006). Antagonists of CCR4 as Immunomodulatory Agents. Curr. Top. Med. Chem..

[57] Stolberg V.R., Martin B., Mancuso P., Olszewski M.A., Freeman C.M., Curtis J.L., Chensue S.W. (2014). Role of CC Chemokine Receptor 4 in Natural Killer Cell Activation during Acute Cigarette Smoke Exposure. Am. J. Pathol..

[58] Zrioual S., Toh M.-L., Tournadre A., Zhou Y., Cazalis M.-A., Pachot A., Miossec V., Miossec P. (2007). IL-17RA and IL-17RC Receptors Are Essential for IL-17A-Induced ELR+ CXC Chemokine Expression in Synoviocytes and Are Overexpressed in Rheumatoid Blood. J. Immunol..

[59] Williams M., Todd I., Fairclough L.C. (2021). The role of CD8 + T lymphocytes in chronic obstructive pulmonary disease: A systematic review. Inflamm. Res..

[60] Hou J., Sun Y. (2020). Role of Regulatory T Cells in Disturbed Immune Homeostasis in Patients with Chronic Obstructive Pulmonary Disease. Front. Immunol..

[61] Barnes P.J. (2008). The cytokine network in asthma and chronic obstructive pulmonary disease. J. Clin. Investig..

[62] Knobloch J., Panek S., Yanik S.D., Jameel K.J., Bendella Z., Jungck D., Bürger P., Bülthoff E., Struck B., Giannakis N. (2019). The monocyte-dependent immune response to bacteria is suppressed in smoking-induced COPD. J. Mol. Med..

[63] Knobloch J., Yakin Y., Körber S., Grensemann B., Bendella Z., Boyaci N., Gallert W.-J., Yanik S.D., Jungck D., Koch A. (2016). Simvastatin requires activation in accessory cells to modulate T-cell responses in asthma and COPD. Eur. J. Pharmacol..

[64] Koch A., Raidl M., Lux M., Müller K., Büning H., Humme S., Erdmann E. (2007). IL-12-induced T-bet expression and IFNγ release in lymphocytes from asthmatics—Role of MAPkinases ERK-1/-2, p38MAPK and effect of dexamethasone. Respir. Med..

[65] Mat Z., Grensemann B., Yakin Y., Knobloch J., Koch A. (2012). Effect of lipoteichoic acid on IL-2 and IL-5 release from T lymphocytes in asthma and COPD. Int. Immunopharmacol..

[66] Knobloch J., Hag H., Jungck D., Urban K., Koch A. (2011). Resveratrol Impairs the Release of Steroid-resistant Cytokines from Bacterial Endotoxin-Exposed Alveolar Macrophages in Chronic Obstructive Pulmonary Disease. Basic Clin. Pharmacol. Toxicol..

